# Preoperative peripheral blood inflammatory markers especially the fibrinogen-to-lymphocyte ratio and novel FLR-N score predict the prognosis of patients with early-stage resectable extrahepatic cholangiocarcinoma

**DOI:** 10.3389/fonc.2022.1003845

**Published:** 2022-10-31

**Authors:** Shijie Li, Xingli Zhang, Changjie Lou, Yuanlong Gu, Juan Zhao

**Affiliations:** ^1^ Department of Interventional Radiology, Harbin Medical University Cancer Hospital, Harbin, China; ^2^ Biotherapy Center, Harbin Medical University Cancer Hospital, Harbin, China; ^3^ Department of Gastrointestinal Medical Oncology, Harbin Medical University Cancer Hospital, Harbin, Heilongjiang, China; ^4^ Department of Interventional Oncology, Taizhou Municipal Hospital, Taizhou, Zhejiang, China

**Keywords:** extrahepatic cholangiocarcinoma, inflammatory markers, neutrophils, FLR, FLR-N score, prognosis

## Abstract

**Background:**

Systemic inflammation is important in the development of extrahepatic cholangiocarcinoma (ECC). The aim of this study was to compare the prognostic power of preoperative peripheral blood inflammatory markers and the novel FLR-N score in patients with resectable ECC.

**Methods:**

A total of 140 patients with resectable ECC and 140 healthy controls (HCs) were recruited for the study. The Mann−Whitney U test was used to evaluate the differences in inflammatory markers between groups. Kaplan−Meier and Cox regression analyses were used to evaluate the prognostic power of preoperative fibrinogen, albumin, prealbumin, bilirubin, neutrophils, lymphocytes, monocytes, platelets, fibrinogen-to-lymphocyte ratio (FLR), fibrinogen-to-albumin ratio (FAR), fibrinogen-to-prealbumin ratio (FPR), neutrophil-to-lymphocyte ratio (NLR), platelet-to-lymphocyte ratio (PLR), monocyte-to-lymphocyte ratio (MLR), FLR-neutrophil (FLR-N) score, and CA19-9 in patients with resectable ECC. Nomogram was developed based on the results of multivariate Cox analyses.

**Results:**

Patients with resectable ECC had significantly higher levels of neutrophils, monocytes, fibrinogen, FLR, FAR, FPR, NLR, PLR, and MLR and lower levels of lymphocytes, albumin, and prealbumin than HCs (all P < 0.01). Albumin, prealbumin, and FPR had a good ability to distinguish between ECC patients with total bilirubin < 34 µmol/L and HCs (AUCs of 0.820, 0.827, and 0.836, respectively). Kaplan−Meier analysis showed that high neutrophil, fibrinogen, FLR, FAR, PLR, MLR, and FLR-N score values were associated with poor survival in patients with resectable ECC. Multivariate analyses indicated that neutrophils (P = 0.022), FLR (P = 0.040), FLR-N score (P < 0.0001), and positive lymph node metastasis (P = 0.016) were independent factors for overall survival (OS). Nomogram were developed to predict OS for patients with ECC.

**Conclusion:**

The prognostic roles of inflammatory markers in patients with resectable ECC were different. The preoperative neutrophil count, FLR and FLR-N score could serve as noninvasive markers for predicting the prognosis of resectable ECC.

## Introduction

Cholangiocarcinoma (CCA) is a rare but highly fatal cancer that arises along intrahepatic or extrahepatic bile ducts; it has very limited treatment options and a total 5-year survival rate of less than 5% (1). The incidence and associated mortality rates of CCA have increased in recent years (2). CCA is classified into intrahepatic CCA (ICC) and extrahepatic CCA (ECC) according to the anatomical location, and ECC is further divided into perihilar CCA (PCC) and distal CCA (DCC) (3). At present, surgical treatment is the preferred therapy for patients with ECC. However, the 5-year survival rates after resection for ECC are approximately 20–40% (4). Therefore, it is crucial to identify effective prognostic indicators for patients with ECC after radical resection.

It is now clear that systemic inflammation is a crucial factor in cancer development and progression (5). Recently, some biomarkers based on systemic inflammation have been verified to predict prognosis in multiple cancers. For example, high levels of fibrinogen, fibrinogen-to-prealbumin ratio (FPR), neutrophil-to-lymphocyte ratio (NLR), and fibrinogen-to-albumin ratio (FAR) could predict poor prognosis in patients with esophageal squamous cell carcinoma (ESCC) (6), resectable gastric cancer (GC) (7), metastatic pancreatic cancer (PC) (8), and triple-negative breast cancer (TNBC) (9). High NLR, platelet-to-lymphocyte ratio (PLR), and FAR values and low lymphocyte-to-monocyte ratio (LMR) values could be prognostic factors for short overall survival (OS) in patients with CCA (10–12). There have been some studies on ICC (13–16); however, very few studies have referred to ECC, PCC (11), and DCC (17, 18). Additionally, no study has yet explored whether inflammatory indicators such as fibrinogen, neutrophil, fibrinogen-to-lymphocyte ratio (FLR), and FPR could be prognostic markers for patients with ECC.

In this study, we first comprehensively assessed the differences in the expression of preoperative circulating inflammatory markers between resectable ECC patients and healthy controls (HCs). Then, we evaluated the prognostic value of inflammatory markers for patients with resectable ECC. Moreover, a new FLR-neutrophil (FLR-N) score based on inflammatory markers was established and was as an independent prognostic factor for OS in patients with resectable ECC.

## Methods

### Patient characteristics

A total of 140 patients with resectable ECC and 140 age- and sex-matched HCs were enrolled from January 2014 to December 2018 at Harbin Medical University Cancer Hospital. The inclusion and exclusion criteria were as follows: a) age over than 18 years; b) no preoperative radiotherapy and/or chemotherapy treatment; c) no acute inflammatory, immune system disease, HIV, hepatitis, blood system disease, or nephropathy; d) pathologically confirmed diagnosis of bile duct adenocarcinomas; e) radical surgical resection; and f) available clinical and follow-up data. Ethical approval was obtained from the Harbin Medical University Cancer Hospital Ethics Committee.

### Clinical data collection

The clinical and pathological information, including sex, age, tumor sites, differentiation, lymph node metastasis, and pathological stage, were collected. The pathological tumor stage (pTNM) was defined based on the American Joint Committee on Cancer (AJCC) 8th edition guidelines. The clinical biochemistry and hematology data within 7 days before surgery were also gathered, including fibrinogen, albumin, prealbumin, bilirubin, neutrophils, lymphocytes, monocytes, platelets, and CA19-9. The patients were followed through the death date or until June 30, 2022. OS was defined as the time from operation to death or the last follow-up.

### Definition of inflammatory marker ratios

FLR, FAR, FPR, NLR, PLR, and MLR were defined as the ratio of the fibrinogen value and lymphocyte value, the ratio of the fibrinogen value and albumin value, the ratio of the fibrinogen value and prealbumin value, the ratio of the neutrophil value and lymphocyte value, the ratio of the platelet value and lymphocyte value, and the ratio of the monocyte value and lymphocyte value, respectively. The FLR-N score was calculated by combining increased FLR and neutrophils; scores of 0 = neither increased, 1= one of them increased, and 2 = both increased.

### Statistical analysis

The data are presented as the mean ± standard deviation (SD). The differences in inflammatory markers in the ECC and HC groups were examined with the Mann−Whitney U test. A *p* value less than 0.05 was considered statistically significant. The diagnostic accuracy of the inflammation indicators and CA19-9 for ECC were evaluated using receiver operating characteristic (ROC) curves and the area under the ROC curve (AUC). The optimal cutoff values of inflammation indicators and CA19-9 were evaluated using the time-dependent ROC (t-ROC) curve according to the maximum Youden index. The associations between inflammatory markers and clinicopathological features were evaluated by the chi-square (χ2) test. The prognostic value of inflammatory markers for OS was assessed using the Kaplan–Meier method and Cox proportional hazards model. Statistical analyses were conducted using SPSS software version 23.0 and GraphPad Prism software version 8.0. The nomogram was developed using the nomogram package in R and concordance indexes(C-index), calibration plots, and decision curve analyses (DCA) were generated to evaluate the performance of the nomogram.

## Results

### Clinicopathologic characteristics of patients with resectable ECC and HCs

Among patients with resectable ECC, the average age was 59.79 ± 8.48 years, and most patients were male (68.6%). A total of 72.1% of patients with total bilirubin ≥ 34 µmol/L presented with clinical obstructive jaundice. The average fibrinogen, albumin, prealbumin, neutrophil, lymphocyte, platelet, and monocyte levels of patients with resectable ECC were 3.67 ± 1.45 g/L, 37.45 ± 4.52 g/L, 198.17 ± 84.64 mg/L, 4.24 ± 1.45×10^9^/L, 1.64 ± 0.55×10^9^/L, 242.73 ± 76.07×10^9^/L, and 0.59 ± 0.21×10^9^/L, respectively. The corresponding average numerical values of HCs were 2.89 ± 0.61 g/L, 43.77 ± 2.37 g/L, 323.88 ± 63.38 mg/L, 3.47 ± 1.03 ×10^9^/L, 1.91 ± 0.50 ×10^9^/L, 224.54 ± 52.57 ×10^9^/L, and 0.38 ± 0.11 ×10^9^/L. The average FLR, FAR, FPR, NLR, PLR, and MLR values were 2.53 ± 1.21, 0.10 ± 0.04, 0.024 ± 0.024, 2.95 ± 1.87, 164.60 ± 78.83, and 0.40 ± 0.19, respectively. The corresponding average numerical values of HCs were 1.63 ± 0.59, 0.066 ± 0.014, 0.009 ± 0.003, 1.92 ± 0.77, 124.54 ± 42.18, and 0.20 ± 0.06. Detailed information on the patient and HC characteristics is listed in **Table 1** and **Supplementary Table 1**.

**Table 1:** Clinicopathologic characteristics and circulating inflammatory markers in patients with ECC and HCs.

Groups	ECC	HCs	P value
Sex			1.000
Male	96 (68.6%)	96 (68.6%)	
Female	44 (31.4%)	44 (31.4%)	
Age			1.000
< 60	63 (45.0%)	63 (45.0%)	
≥60	77 (55.0%)	77 (55.0%)	
Bilirubin			
Total bilirubin ≥ 34umol/L	101 (72.1%)		
Total bilirubin < 34umol/L	39 (27.9%)		
Location			
Perihilar	83 (59.3%)		
Distal	57 (40.7%)		
Differentiation			
High and Moderate	82 (58.6%)		
Poor	58 (41.4%)		
Lymph nodes metastasis			
Yes	27 (15.0%)		
No	113 (85.0%)		
TNM stage			
I-II	120 (85.7%)		
III	20 (14.3%)		
Fibrinogen (g/L, Mean ± SD)	3.67±1.45	2.89±0.61	< 0.001
Albumin (g/L, Mean ± SD)	37.45±4.52	43.77±2.37	< 0.001
Prealbumin (mg/L, Mean ± SD)	198.17±84.64	323.88±63.38	< 0.001
Neutrophil (×10^9^/L, Mean ± SD)	4.24±1.45	3.47±1.03	< 0.001
Lymphocyte (×10^9^/L, Mean ± SD)	1.64±0.55	1.91±0.50	< 0.001
Platelet (×10^9^/L, Mean ± SD)	242.73±76.07	224.54±52.57	0.005
Monocyte (×10^9^/L, Mean ± SD)	0.59±0.21	0.38±0.11	< 0.001
FLR (Mean ± SD)	2.53±1.21	1.63±0.59	< 0.001
FAR (Mean ± SD)	0.10±0.04	0.066±0.014	< 0.001
FPR (Mean ± SD)	0.024±0.024	0.009±0.003	< 0.001
NLR (Mean ± SD)	2.95±1.87	1.92±0.77	< 0.001
PLR (Mean ± SD)	164.60±78.83	124.54±42.18	< 0.001
MLR (Mean ± SD)	0.40±0.19	0.20±0.06	< 0.001

ECC, extrahepatic cholangiocarcinoma; HCs, healthy controls; FLR, fibrinogen-to-lymphocyte ratio; FAR, fibrinogen-to-albumin ratio; FPR, fibrinogen-to-prealbumin ratio; NLR, neutrophil-to-lymphocyte ratio; MLR, monocyte-to-lymphocyte ratio; PLR, platelet-to-lymphocyte ratio.

### Inflammatory marker values of patients with resectable ECC and HCs

**Figure 1:**
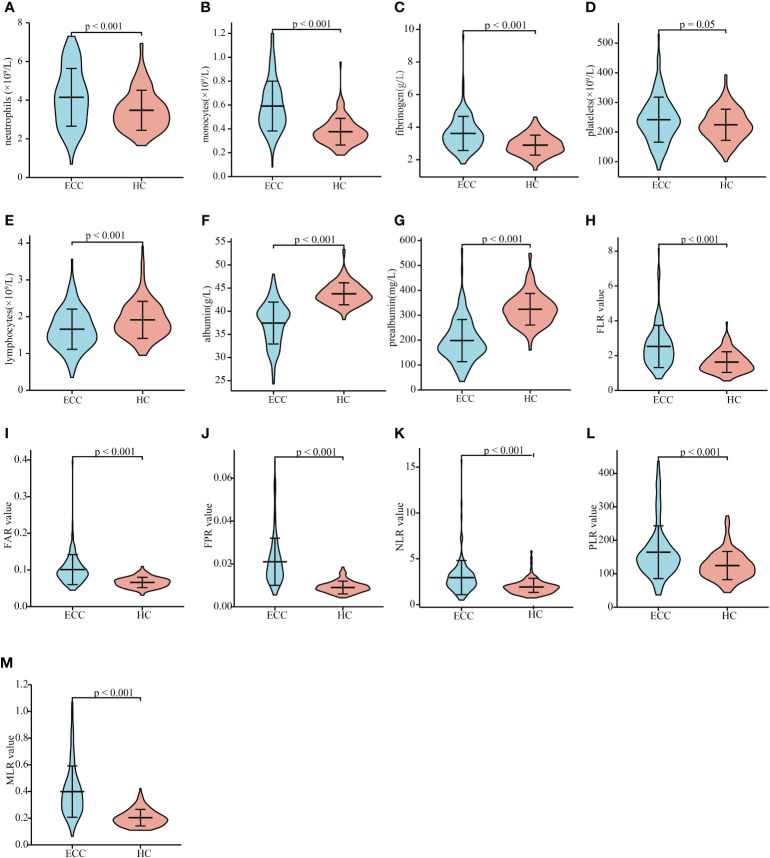
Inflammation indicator levels in patients with resectable ECC and HCs. The circulating neutrophil **(A)**, monocyte **(B)**, fibrinogen **(C)**, platelet **(D)**, lymphocyte **(E)**, albumin **(F)**, and prealbumin **(G)** levels in patients with ECC and HCs. FLR **(H)**, FAR **(I)**, FPR **(J)**, NLR **(K)**, PLR **(L)**, and MLR **(M)** values in patients with ECC and HCs. ECC, extrahepatic cholangiocarcinoma; HC, heathy controls; FLR, fibrinogen-to-lymphocyte ratio; FAR, fibrinogen-to-albumin ratio; FPR, fibrinogen-to-prealbumin ratio; NLR, neutrophil-to-lymphocyte ratio; PLR, platelet-to-lymphocyte ratio; MLR monocyte-to-lymphocyte ratio.

We then compared the levels of inflammatory markers between ECC patients and HCs. Patients with ECC had significantly higher neutrophil, monocyte, and fibrinogen levels and lower lymphocyte, albumin, and prealbumin levels than HCs (**Figures 1A-1F**, all P < 0.01). The levels of platelets were slightly elevated in ECC, but the difference between the two groups was not statistically significant (**Figure 1G**, P = 0.05). Patients with ECC had significantly higher FLR, FAR, FPR, NLR, PLR, and MLR values than HCs (**Figures 1H–1M** all P < 0.01). The above results showed that circulating inflammatory markers changed in the early stage of cancer. In addition, we applied ROC analysis to assess the capabilities of inflammatory markers to discriminate between ECC and HCs. Monocyte, albumin, prealbumin, FAR, FPR, MLR, and CA19-9 can be used to discriminate ECC from HCs (**Supplementary Figures 1A, 1B**; AUCs of 0.837, 0.910, 0.891, 0.840, 0.915, 0.872, and 0.895, respectively). Then, we divided the patients into two subgroups based on total bilirubin < 34 and ≥ 34 µmol/L. Albumin, prealbumin, and FPR had a good ability to distinguish between ECC patients with total bilirubin < 34 µmol/L and HCs (**Supplementary Figures 1C, 1D**; AUCs of 0.820, 0.827, and 0.836, respectively). The results demonstrated that albumin, prealbumin, and FPR might serve as auxiliary diagnostic indicators for patients at their first visit.

### Optimal cutoff values of inflammatory markers in patients with ECC

The best cutoff values of inflammation indicators for 5-year OS were calculated using t-ROC curves. The best cutoff values of preoperative neutrophils, lymphocytes, monocytes, platelets, fibrinogen, albumin, prealbumin, FLR, FAR, FPR, NLR, MLR, PLR, and CA19-9 were 3.68, 1.57, 0.96, 255, 3.4, 42.1, 187, 2.09, 0.082, 0.013, 2.66, 0.35, 125.82, and 98.34, respectively (**Table 2**). We divided the patients with ECC into either low- or high-value group based on the optimum cutoff value. As shown in **Table 3**, elevated FLR (≥ 2.09) and FAR (≥ 0.082) were significantly associated with distal cholangiocarcinoma (P = 0.012 and 0.003), and high PLR (≥125.82) was associated with lymph node metastasis (P = 0.022).

**Table 2:** ROC analyses of the inflammatory marker values for 5-year OS in patients with ECC.

Markers	Cutoff point	AUC (95% CI)	Sensitivity	Specificity
FLR	2.09	0.674 (0.554-0.795)	0.697	0.817
FAR	0.082	0.652 (0.528-0.775)	0.762	0.542
FPR	0.013	0.497 (0.373-0.620)	0.777	0.083
NLR	2.66	0.712 (0.598-0.825)	0.792	0.866
MLR	0.35	0.544 (0.408-0.680)	0.581	0.583
PLR	125.82	0.616 (0.486-0.745)	0.745	0.500
Neutrophils	3.68	0.681 (0.566-0.797)	0.723	0.625
Lymphocytes	1.57	0.460 (0.329-0.590)	0.438	0.417
Monocytes	0.96	0.494 (0.362-0.627)	0.052	0.833
Platelets	255	0.595 (0.472-0.717)	0.453	0.792
Fibrinogen	3.4	0.687 (0.569-0.806)	0.678	0.708
Albumin	42.1	0.489 (0.359-0.620)	0.091	0.833
Prealbumin	187	0.594 (0.475-0.713)	0.524	0.708
CA199	98.34	0.560 (0.438-0.682)	0.602	0.667

ROC, receiver operating characteristic; OS, overall survival; AUC, area under the ROC curve; CI, confidence interval; FLR, fibrinogen-to-lymphocyte ratio; FAR, fibrinogen-to-albumin ratio; FPR, fibrinogen-to-prealbumin ratio; NLR, neutrophil-to-lymphocyte ratio; MLR, monocyte-to-lymphocyte ratio; PLR, platelet-to-lymphocyte ratio.

**Table 3:** Correlation between the preoperative inflammatory markers and clinicopathological features in patients with ECC.

Groups	Neutrophil [n (%)]		P	Fibrinogen [n (%)]		P	FLR [n (%)]		P	FAR [n (%)]		P	NLR [n (%)]		P	PLR [n (%)]		P	MLR [n (%)]		P
	≥3.68	<3.68		≥3.40	<3.40		≥2.09	<2.09		≥0.082	<0.082		≥2.66	<2.66		≥125.82	<125.82		≥0.35	<0.35	
	86	54		80	60		83	57		96	44		71	69		93	47		73	67	
**Sex**			0.257			0.248			0.130			0.043			0.399			0.044			0.151
Male	62(72.1)	34(63.0)		58(72.5)	38(63.3)		61(73.5)	35(61.4)		71(74.0)	25(56.8)		51(71.8)	45(65.2)		69(74.2)	27(57.4)		54(74.0)	42(62.7)	
Female	24(27.9)	20(37.0)		22(27.5)	22(36.7)		22(26.5)	22(38.6)		25(26.0)	19(43.2)		20(28.2)	24(34.8)		24(25.8)	20(42.6)		19(26.0)	25(37.3)	
**Age**			0.917			0.731			0.641			0.770			0.316			0.257			0.020
≤60	39(45.3)	24(44.4)		37(46.3)	26(43.3)		36(43.4)	27(47.4)		44(45.8)	19(43.2)		29(40.8)	34(49.3)		45(48.4)	18(38.3)		26(25.6)	37(55.2)	
>60	47(54.7)	30(55.6)		43(53.8)	34(56.7)		47(56.6)	30(52.6)		52(54.2)	25(56.8)		52(59.2)	35(50.7)		48(51.6)	29 (61.7)		47(64.4)	30(44.8)	
**Location**			0.156			0.882			0.012			0.003			0.707			0.061			0.660
Perihilar	55(64.0)	28(51.9)		47(58.8)	36(60.0)		42(50.6)	41(71.9)		49(51.0)	34(77.3)		41(57.7)	42(60.9)		50(53.8)	33(70.2)		42(57.5)	41(61.2)	
Distal	31(36.0)	26(48.1)		33(41.3)	24(40.0)		41(49.4)	16(28.1)		47(49.0)	10(22.7)		30(4.3)	27(39.1)		43(46.2)	14(29.8)		31(42.5)	26(38.8)	
**Differentiation**			0.896			0.181			0.573			0.118			0.627			0.864			0.669
High	50(58.1)	32(59.3)		43(53.8)	39(65.0)		47(56.6)	35(61.4)		52(54.2)	30(68.2)		43(60.6)	39(56.5)		54(58.1)	28(59.6)		44(60.3)	38(56.7)	
Poor	36(41.9)	22(40.7)		37(46.3)	21(35.0)		36(43.4)	22(38.6)		44(45.8)	14(31.8)		28(39.4)	30(43.5)		39(41.9)	19(40.4)		29(39.7)	29(43.3)	
**Lymph nodes**			0.288			0.266			0.998			0.493			0.575			0.022			0.373
+	19(22.1)	8(14.8)		18(22.5)	9(15.0)		16(19.3)	11(19.3)		20(20.8)	7(15.9)		15(21.1)	12(17.4)		23(24.7)	4(8.5)		12(16.4)	15(22.4)	
–	67(77.9)	46(85.2)		62(77.5)	51(85.0)		67(80.7)	46(80.7)		76(79.2)	37(84.1)		56(78.9)	57(82.6)		70(75.3)	43(91.5)		61(83.6)	52(77.6)	
**TNM stage**			0.178			0.081			0.547			0.234			0.168			0.165			0.447
I	71(82.6)	49(90.7)		65(81.3)	55(91.7)		70(84.3)	50(87.7)		80(83.3)	40(90.9)		58(81.7)	62(89.9)		77(82.8)	43(91.5)		61(83.6)	59(88.1)	
II	15(17.4)	5(9.3)		16(18.8)	5(8.3)		13(15.7)	7(12.3)		16(16.7)	4(9.1)		13(18.3)	7(10.1)		16(17.2)	4(8.5)		12(16.4)	8(11.9)	

### Inflammatory markers were associated with the OS of ECC

**Figure 2:**
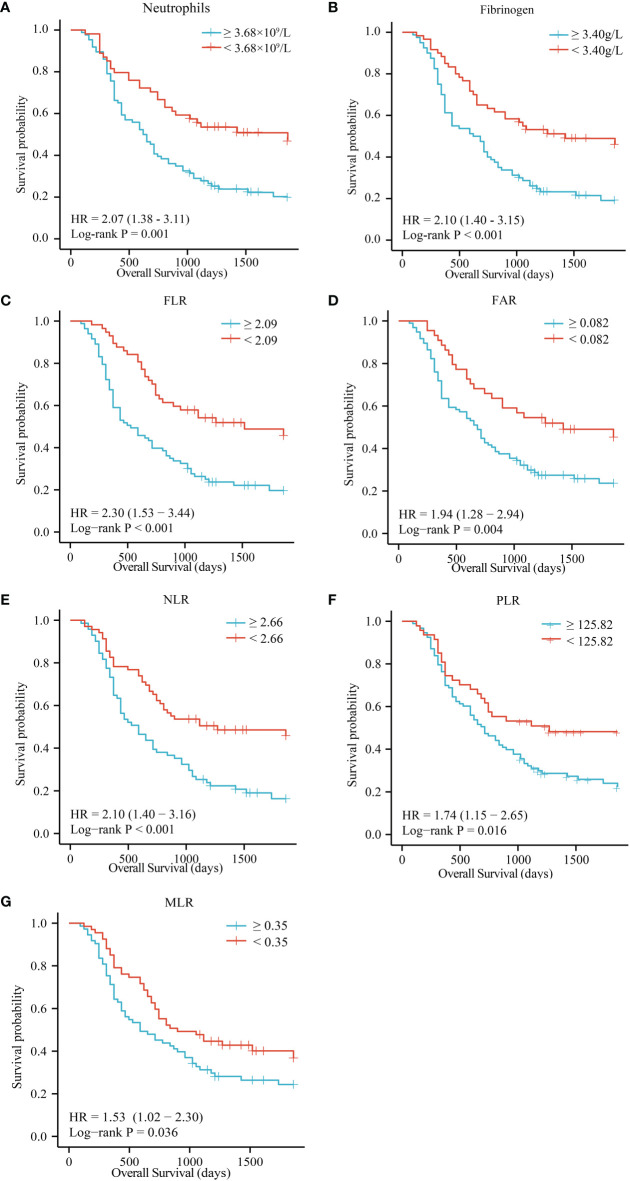
OS of patients with resectable ECC based on neutrophils **(A)**, fibrinogen **(B)**, FLR **(C)**, FAR **(D)**, NLR **(E)**, PLR **(F)**, and MLR **(G)**. OS, overall survival; FLR, fibrinogen-to-lymphocyte ratio; FAR, fibrinogen-to-albumin ratio; NLR, neutrophil-to-lymphocyte ratio; PLR, platelet-to-lymphocyte ratio; MLR monocyte-to-lymphocyte ratio.

High preoperative peripheral blood neutrophils and fibrinogen were correlated with a short OS in patients with ECC (**Figures 2A, 2B**, P = 0.001 and P < 0.001). There were no significant associations between lymphocytes, monocytes, platelets, albumin, and prealbumin and prognosis (**Supplementary Figures 2A-2E**, P = 0.079, 0.452, 0.146, 0.358, and 0.431). Meanwhile, high FLR, FAR, NLR, PLR, and MLR were associated with poor OS in patients with ECC (**Figures 2C-2G**, P < 0.001, P = 0.004, P < 0.001, P = 0.016, and P = 0.036, respectively). However, FPR and CA19-9 levels were not correlated with OS (**Supplementary Figures 2 F-2G**, P = 0.716 and P = 0.129). We further conducted univariate and multivariate survival analyses for baseline characteristics and inflammation indicators using Cox regression models. As shown in **Table 4**, univariate analysis showed that lymph node metastasis (HR = 2.23, 95% CI: 1.40–3.56, P = 0.001), poor differentiation (HR = 1.68, 95% CI: 1.12–2.53, P = 0.012), TNM stage III (HR = 1.78, 95% CI: 1.04–3.06, P = 0.037), higher neutrophils (HR = 2.09, 95% CI: 1.33–3.28, P = 0.001), higher fibrinogen (HR = 2.12, 95% CI: 1.38–3.27, P = 0.001), higher FLR (HR = 2.32, 95% CI: 1.50–3.60, P < 0.0001), higher FAR (HR = 1.96, 95% CI: 1.22–3.14, P = 0.005), higher NLR (HR = 2.12, 95% CI: 1.40–3.23, P < 0.0001), higher PLR (HR = 1.75, 95% CI: 1.10–2.78, P = 0.019), and higher MLR (HR = 1.54, 95% CI: 1.02–2.31, P = 0.040) were significant poor prognostic factors for OS. Multivariate analysis showed that lymph node metastasis (HR = 2.17, 95% CI: 1.16–4.06, P = 0.016), neutrophils (HR = 1.89, 95% CI: 1.10–3.27, P = 0.022), and FLR (HR = 2.02, 95% CI: 1.03–3.94, P = 0.040) were significant independent predictors of OS.

**Table 4:** Univariate and multivariate analysis of OS in patients with ECC.

	Univariate analysis		Multivariate analysis	
Variable	HR (95% CI)	P	HR (95% CI)	P
Sex (female vs. male)	0.93 (0.60-1.45)	0.760		
Age (≤ 60 vs. > 60)	1.05 (0.70-1.58)	0.799		
Location (distal vs. perihilar)	0.72 (0.47-1.11)	0.134		
Lymph nodes metastasis (yes vs. no)	2.23 (1.40-3.56)	0.001	2.17 (1.16-4.06)	0.016
Differentiation (poor vs. well)	1.68 (1.12-2.53)	0.012	1.55 (0.97-2.46)	0.065
Stage (III vs. I-II)	1.78 (1.04-3.06)	0.037	1.09 (0.56-2.12)	0.808
CA199(≥ 98.34 vs. < 98.34)	0.73 (0.48-1.10)	0.127		
Neutrophil (≥ 3.68 vs. < 3.68)	2.09 (1.33-3.28)	0.001	1.89 (1.10-3.27)	0.022
Fibrinogen (≥ 3.40 vs. < 3.40)	2.12 (1.38-3.27)	0.001	1.46 (0.77-2.79)	0.248
FLR (≥ 2.09 vs. < 2.09)	2.32 (1.50-3.60)	< 0.0001	2.02 (1.03-3.94)	0.040
FAR (≥ 0.082 vs. < 0.082)	1.96 (1.22-3.14)	0.005	1.06 (0.53-2.15)	0.860
FPR (≥ 0.013 vs. < 0.013)	0.92 (0.57-1.488)	0.720		
NLR (≥ 2.66 vs. < 2.66)	2.12 (1.40-3.23)	< 0.0001	1.05 (0.55-1.99)	0.880
PLR (≥ 125.82 vs. < 125.82)	1.75 (1.10-2.78)	0.019	1.04 (0.59-1.81)	0.900
MLR (≥ 0.35 vs. < 0.35)	1.54 (1.02-2.31)	0.040	1.23 (0.75-2.03)	0.408
FLR-N (2 vs. 0-1)	2.714 (1.728-4.265)	< 0.0001	2.69 (1.76-4.09)	< 0.0001

ECC, extrahepatic cholangiocarcinoma; OS, overall survival; FLR, fibrinogen-to-lymphocyte ratio; FAR, fibrinogen-to-albumin ratio; FPR, fibrinogen-to-prealbumin ratio; NLR, neutrophil-to-lymphocyte ratio; MLR, monocyte-to-lymphocyte ratio; PLR, platelet-to-lymphocyte ratio.

### Subgroup analysis

**Figure 3:**
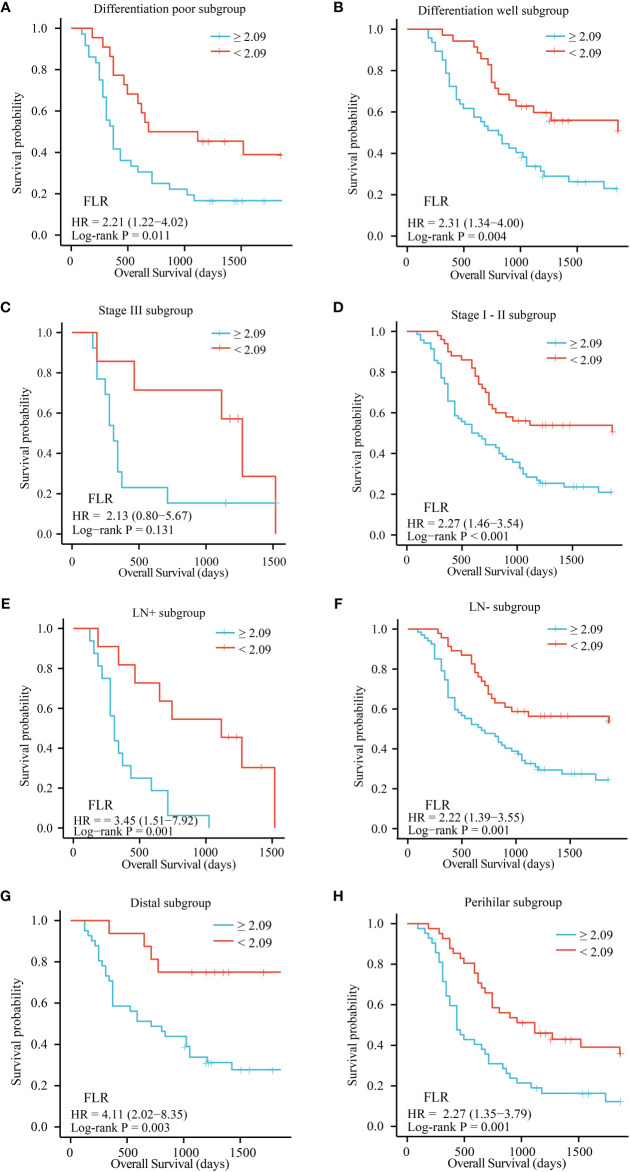
OS of patients with resectable ECC based on the optimal cutoff value of FLR in each subgroup. **(A)** Differentiation poor subgroup; **(B)** Differentiation well subgroup; **(C)** Stage III subgroup; **(D)** Stage I-II subgroup; **(E)** LN+ subgroup; **(F)** LN- subgroup; **(G)** Distal subgroup; **(H)** Perihilar subgroup. OS, overall survival; FLR, fibrinogen-to-lymphocyte ratio; LN-, lymph node metastasis negative; LN+, lymph node metastasis positive.

The patients were divided into separate subgroups according to tumor site, differentiation, lymph node metastasis, and TNM stage to explore the prognostic significance of FLR and neutrophils in ECC. As shown in **Figure 3**, a high FLR value was correlated with poor prognosis in the well and poor differentiation subgroups (P = 0.011 and P = 0.004), stage I-II subgroups (P = 0.005 and P < 0.001), lymph node metastasis positive and negative subgroups (P = 0.001 and P = 0.001), and distal and perihilar tumor subgroups (P = 0.003 and P = 0.001). High neutrophil values were correlated with poor prognosis in the well-differentiated and poorly differentiated subgroups (P = 0.037 and P = 0.008), stage I-II subgroups (P = 0.001), lymph node metastasis negative subgroup (P = 0.005), and distal and perihilar tumor subgroups (P = 0.015 and P = 0.027) (**Supplementary Figure 3A-3H**).

### The FLR-N score predicted prognosis better than other inflammatory markers

**Figure 4:**
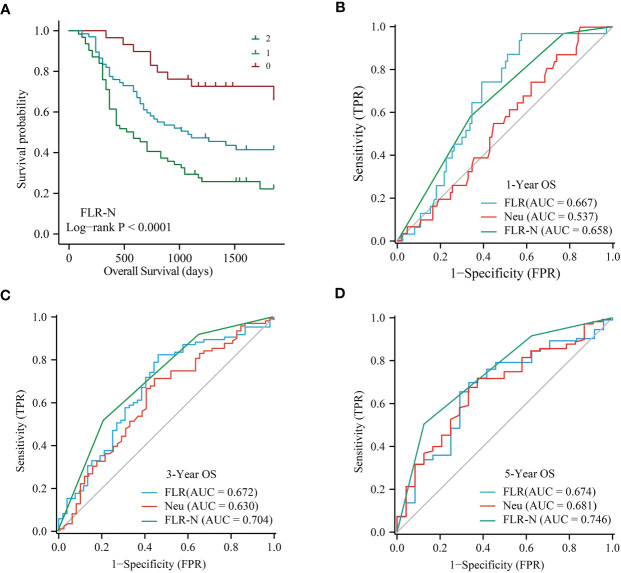
Predictive power of FLR-N for patients with resectable ECC. **(A)** Kaplan−Meier curves for OS based on FLR-N. B, ROC curve analyses of FLR, neutrophils, and FLR-N for 1-year **(B)**, 3-year **(C)**, and 5-year **(D)** OS in patients with resectable ECC. ROC, receiver operating characteristic; AUC, area under the ROC curve; OS, overall survival; FLR, fibrinogen-to-lymphocyte ratio; FLR-N, FLR + neutrophils.

Given that the FLR and neutrophils were independent prognostic factors for ECC by multivariate analysis, we constructed the FLR-N score by combining the FLR with neutrophils. A high FLR-N score was demonstrated to be significantly associated with short OS (**Figure 4A**, P < 0.0001). Multivariate survival analysis based on lymph node metastasis, TNM stage, and differentiation indicated that the FLR-N score was a significant independent predictor of OS (**Table 4**, HR = 2.69, 95% CI: 1.76-4.09, P < 0.0001). In addition, we compared FLR, neutrophils, and FLR-N score in terms of their ability to predict prognosis in ECC. FLR had the best prognostic power for 1-year OS, with an AUC of 0.667, compared with FLR-N, with an AUC of 0.658, and neutrophils, with an AUC of 0.537 (**Figure 4B**). The AUCs of the FLR-N score for 3-year OS and 5-year OS were both greater than those of FLR and neutrophils (**Figures 4C, 4D**, AUC for 3-year OS 0.704 vs. 0.672 and 0.630; AUC for 5-year OS 0.746 vs. 0.674 and 0.681). This result showed that the prognostic power of FLR-N was greater than that of FLR and neutrophils alone. In addition, a high FLR-N score was associated with poor prognosis in the well and poor differentiation subgroups (P = 0.015 and P < 0.001), stage I-II subgroups (P < 0.001), lymph node metastasis positive and negative subgroups (P = 0.001 and P = 0.001), and distal and perihilar tumor subgroups (P = 0.003 and P = 0.001) (**Supplementary Figure 3I-3P**). In addition, since FLR-N was a categorical variable, we also made C index would be a better way to assess the prognostic capability. The C-index of FLR-N was 0.643 for OS (**Supplementary Figure 4A**). Short OS was found in patients with high FLR-N score in low FPR group (p = 0.023, **Supplementary Figure 4B**).

### Construction and evaluation of prognostic models

**Figure 5:**
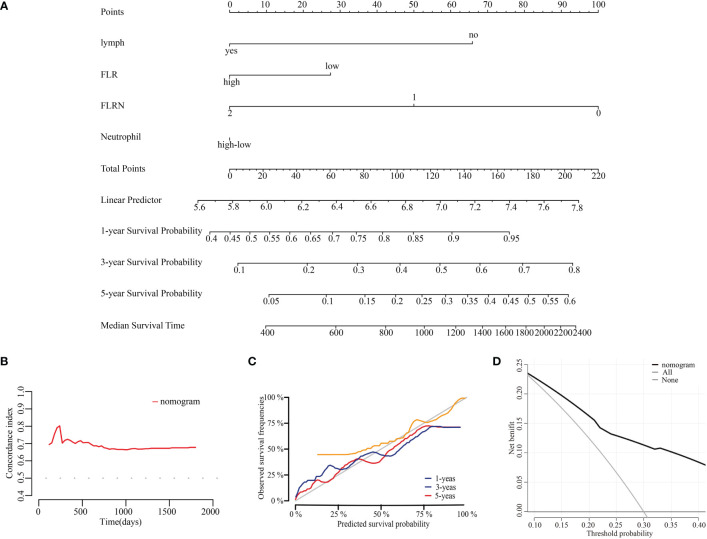
Construction and evaluation of prognostic nomogram. **(A)** Nomogram for prognosis of ECC. **(B)** C-index curves of the model at different times. **(C)** Calibration curves of the model at different times. **(D)** DCA curves of the model.

Based on the results of multivariate Cox analyses, we developed a nomogram to assess OS at 1-, 3-, and 5-years for ECC, in which each signature was assigned points according to its risk contribution to OS (**Figure 5A**). Moreover, the C-index of the nomogram for OS were 0.671 in patients with ECC (**Figure 5B**). The calibration curves for this nomogram at 1-, 3-, and 5-years were good (**Figure 5C**). The DCA indicated that if the threshold probability is between 0.08 ~ 0.85, the model showed better clinical benefit (**Figure 5D**).

## Discussion

Systematic inflammation is closely related to cancer development, and inflammation may already exist at early stages of tumorigenesis (19). Cancer cells produce granulocyte colony-stimulating factor (G-CSF), which promotes the upregulation of circulating neutrophils (20). Elevated neutrophils can secrete interleukin (IL)-1, IL-10, and vascular endothelial growth factor (VEGF), thereby inhibiting lymphocytes and natural killer cells, stimulating angiogenesis and promoting tumor progression (21). Lymphocyte reduction may depress lymphocyte-mediated antitumor cellular immune responses (22). Platelets can activate the TGFβ/Smad and NF-κB signaling pathways in cancer cells, thereby promoting the transition of cancer cells to a more aggressive mesenchymal-like phenotype and accelerating cancer metastasis (23). Platelets promote cancer metastasis by releasing adenine nucleotides, which induce endothelial barrier opening to allow the transendothelial migration of cancer cells and thereby promote cancer cell extravasation (24). Peripheral monocytes are positively associated with tumor-associated macrophages (TAMs), which are associated with tissue remodeling, immune regulation, and angiogenesis, enhancing tumor cell migration and immune evasion (25). Systemic inflammation promotes the release of fibrinogen, which promotes tumor cell proliferation and metastasis by participating in extracellular matrix formation and inducing epithelial–mesenchymal transition *via* the p-AKT/p-mTOR pathway and IL-6 synthesis (26–28). Albumin inhibits tumor progression by stabilizing DNA replication and enhancing immune responses (29). In this study, patients with ECC had increased peripheral blood neutrophils, monocytes, and fibrinogen and reduced lymphocytes, albumin, and prealbumin compared to HCs. Similarly, inflammation indicator ratios, including FLR, FAR, FPR, NLR, PLR, and MLR, were increased in ECC patients compared to HCs. We found no significant difference in platelet count between ECC and HC, presumably because patients with ECC were at an early stage. The ROC curve analysis showed that monocytes, albumin, prealbumin, FAR, FPR, and MLR had good abilities to discriminate patients with ECC from HCs (all AUCs > 0.8). A subgroup analysis showed that in patients with no clinical jaundice (total bilirubin < 34 µmol/L), albumin, prealbumin, and FPR could distinguish patients with ECC from HCs. Therefore, albumin, prealbumin, and FPR abnormalities may suggest ECC for patients with abdominal discomfort at the initial visit. Regrettably, we did not include patients with benign bile duct diseases, such as primary biliary cirrhosis, primary sclerosing cholangitis or other malignancies, in this study. Further research is needed to determine the differential diagnostic values of inflammation indicators.

Inflammatory indicators have been demonstrated by multiple studies to be strong prognostic indicators for the survival of various malignancies. Wang’s study showed that patients with gastric cancer (GC) who had a higher peripheral blood neutrophil percentage had lower OS rates (30). Jiang’s study confirmed that decreased peripheral blood monocyte count (<0.665 × 10^9^/L) was significantly related to longer OS in metastatic nasopharyngeal carcinoma (NPC) (31). He’s study showed that a high percentage of lymphocytes predicted good prognosis in NPC (32). Dai’s study confirmed that elevated levels of fibrinogen predicted poor outcomes in patients with HBV-related hepatocellular carcinoma (HCC) (33). Liu’s study showed that hypoalbuminemia is an independent poor prognostic indicator in patients with nonmetastatic breast cancer (34). Jia’s study showed that a high preoperative level of serum prealbumin could be a poor prognostic factor for HCC (35). Elevated NLR, FAR, FPR, PLR, and MLR levels were related to the poor prognosis of patients with a variety of malignant tumors, including colorectal cancer (CRC) (36), GC (7), PC (8), ICC (10), and HCC (37). Fan’s study showed that a higher FLR was significantly related to poor OS and DFS for ESCC (38). In ECC, Ji’s study showed that an increased NLR was related to poor prognosis for patients with DCC (39), and Lin’s study showed that a high LMR could predict poor prognosis in patients with PCC after R0 radical resection (11). However, these were not studies comparing the prognostic power of inflammatory indicators for ECC. To our knowledge, this is the first study to evaluate the differences in the ability of fibrinogen, albumin, prealbumin, bilirubin, neutrophils, lymphocytes, monocytes, platelets, FLR, FAR, FPR, NLR, PLR, MLR, and CA19-9 to predict the prognosis of ECC patients. We found that high levels of neutrophils, fibrinogen, FLR, FAR, NLR, PLR, and MLR were related to poor prognosis, and FLR and neutrophils were more powerful than other inflammation indicators for patients with resectable ECC. These findings support previous findings that inflammation indicators are effective markers for predicting the prognosis of ECC. Moreover, these inflammation indicators also enriched the existing predictive markers for the prognosis of ECC.

In subgroup analysis, we found that high FLR and FLR-N were related to short OS in patients with ECC across lymph node metastasis yes/no, differentiation well/poor, tumor site perihilar/distal and stage I-II subgroups but were not associated with OS in stage III subgroups. For patients with stage III and lymph node metastasis, neutrophils had no correlation with OS. These results demonstrated that FLR and the FLR-N score were highly effective predictors of patients with early-stage resectable ECC.

Interestingly, it has been reported that the combination of multiple indicators has better prognostic value than their individual use. Tang’s study showed that the combination of FAR, FPR, and CEA could better predict GC patient OS (7). Lwasaki’s study showed that combined fibrinogen and NLR was a potential prognostic indicator in patients with resectable NSCLC (40). In this study, the FLR-N score was superior to FLR and neutrophils alone and could be an independent prognostic factor for early-stage resectable ECC. The nomogram was constructed to predict the 1-, 3-, and 5-year OS of resectable ECC. The discrimination and calibration of nomogram showed that the nomogram was valuable, and the variables in the nomogram were easy to be obtained, which makes it easy to convert it into allowing feasible translation into clinical applications.

There are several limitations in our study. First, benign bile duct diseases and ampullary/periampullary-duodenal cancers were not included in this study. Second, this study is a single-center trial with a limited sample size, which may not be representative of other cohorts. Third, adjuvant chemotherapy could have affected the survival outcomes. Hence, further multicentric studies and large sample sizes are needed to validate the conclusions.

## Conclusion

In summary, peripheral blood inflammatory marker changes were apparent at an early stage in patients with ECC, and inflammatory markers, especially albumin, prealbumin, and FPR, could be able to discriminate patients with early-stage resectable ECC from HCs. In addition, preoperative peripheral blood inflammatory markers, especially FLR and the FLR-N score, can serve as noninvasive predictors of OS for patients with resectable ECC. Nomogram can help clinicians identify patients with poor prognosis.

## Data availability statement

The original contributions presented in the study are included in the article/**Supplementary Material**. Further inquiries can be directed to the corresponding author.

## Ethics statement

The studies involving human participants were reviewed and approved by the Harbin Medical University Cancer Hospital Ethics Committee (KY2022-14). The patients/participants provided their written informed consent to participate in this study.

## Authors contributions

SL, JZ, and YG conceived and designed the study. XZ and CL collected the samples and worked on the experiment. SL, XZ, and CL performed statistical analysis and wrote the manuscript. JZ and YG reviewed and edited the manuscript. All authors contributed to the article and approved the submitted version.

## Funding

This work was supported by the Health Commission of Heilongjiang (grant number 2019–060), Haiyan Fund Project of Harbin Medical University Cancer Hospital (JJZD2020-03), Zhejiang Provincial Natural Science Foundation of China (grant no. LGC22H160012), and Zhejiang Provincial Health Planning Commission Fund (grant no. 2020389903).

## Conflict of interest

The authors declare that the research was conducted in the absence of any commercial or financial relationships that could be construed as a potential conflict of interest.

## Publisher’s note

All claims expressed in this article are solely those of the authors and do not necessarily represent those of their affiliated organizations, or those of the publisher, the editors and the reviewers. Any product that may be evaluated in this article, or claim that may be made by its manufacturer, is not guaranteed or endorsed by the publisher.
