# Supplementary material for: Preoperative peripheral blood inflammatory markers especially the fibrinogen-to-lymphocyte ratio and novel FLR-N score predict the prognosis of patients with early-stage resectable extrahepatic cholangiocarcinoma
Source: Front Oncol. 2022 Oct 31;12:1003845. doi: 10.3389/fonc.2022.1003845 (PMC9659886; doi:10.3389/fonc.2022.1003845)
Supplement: Supplementary file 1 [file Table_1.docx]

| Supplementary Table 1 The baseline information of 140 patients with extrahepatic cholangiocarcinoma (ECC) | | | | | | | | | | |
| --- | --- | --- | --- | --- | --- | --- | --- | --- | --- | --- |
| Patients | Follow up time | Status | Age | Sex | Location | Differentiation | Lymph | Stage | Amount of blood loss of operation (ml) | Chemotherapy after operation |
| p1 | 806 | die | 44 | male | distal | well | no | IA | 300 | yes |
| p2 | 1054 | die | 68 | male | distal | well | no | IB | 500 | no |
| p3 | 1860 | live | 63 | male | distal | well | no | IIA | 800 | yes |
| p4 | 1860 | live | 66 | male | distal | well | no | IIA | 200 | yes |
| p5 | 217 | die | 61 | female | perihilar | poor | yes | IIB | 1400 | yes |
| p6 | 310 | die | 40 | male | perihilar | poor | no | IA | 500 | yes |
| p7 | 310 | die | 52 | male | perihilar | poor | no | IA | 150 | yes |
| p8 | 1860 | live | 50 | female | perihilar | well | no | IA | 500 | yes |
| p9 | 372 | die | 74 | female | distal | well | no | IIA | 200 | no |
| p10 | 372 | die | 63 | male | distal | poor | no | IIA | 100 | yes |
| p11 | 1860 | live | 57 | female | perihilar | well | no | IA | 150 | yes |
| p12 | 1860 | live | 30 | female | perihilar | poor | no | IB | 300 | yes |
| p13 | 1860 | live | 65 | female | distal | well | no | IB | 400 | yes |
| p14 | 1860 | live | 46 | male | distal | poor | no | IB | 600 | yes |
| p15 | 1860 | live | 53 | male | distal | well | no | IIA | 500 | yes |
| p16 | 713 | die | 57 | male | perihilar | poor | no | IB | 200 | yes |
| p17 | 372 | die | 56 | male | distal | poor | no | IA | 200 | yes |
| p18 | 1178 | die | 54 | male | perihilar | well | no | IIB | 800 | yes |
| p19 | 155 | die | 63 | female | perihilar | poor | yes | IIB | 250 | yes |
| p20 | 186 | die | 65 | male | perihilar | well | yes | III | 100 | no |
| p21 | 744 | die | 54 | female | perihilar | well | yes | IIB | 200 | yes |
| p22 | 93 | die | 69 | male | perihilar | poor | no | IA | 50 | no |
| p23 | 1860 | live | 72 | male | distal | well | no | IIA | 400 | yes |
| p24 | 1860 | live | 47 | female | perihilar | poor | no | IA | 100 | yes |
| p25 | 589 | die | 51 | female | perihilar | well | no | II | 800 | yes |
| p26 | 1860 | live | 68 | male | perihilar | poor | no | IB | 1200 | yes |
| p27 | 868 | die | 60 | male | perihilar | poor | no | II |  | yes |
| p28 | 341 | die | 63 | female | perihilar | well | no | IA | 100 | yes |
| p29 | 713 | die | 64 | female | perihilar | poor | yes | III | 400 | yes |
| p30 | 248 | die | 55 | female | perihilar | well | no | IIB | 500 | yes |
| p31 | 310 | die | 52 | female | distal | well | yes | III | 400 | yes |
| p32 | 1860 | live | 51 | male | distal | well | no | IIB | 800 | yes |
| p33 | 1736 | die | 59 | male | perihilar | well | no | IB | 1000 | yes |
| p34 | 434 | die | 46 | male | perihilar | well | no | IIB | 1500 | yes |
| p35 | 744 | die | 64 | male | perihilar | well | no | IIA | 50 | yes |
| p36 | 1860 | live | 64 | female | perihilar | well | no | IB | 100 | yes |
| p37 | 1054 | die | 68 | male | distal | well | no | IA | 100 | yes |
| p38 | 372 | die | 50 | male | perihilar | poor | no | IA | 200 | yes |
| p39 | 434 | die | 57 | male | perihilar | well | no | IA | 400 | yes |
| p40 | 341 | die | 66 | male | distal | well | yes | III | 300 | yes |
| p41 | 248 | die | 68 | female | distal | poor | no | IB | 300 | yes |
| p42 | 372 | die | 55 | female | distal | poor | yes | III | 1000 | yes |
| p43 | 1271 | die | 72 | female | perihilar | well | yes | III | 400 | no |
| p44 | 279 | die | 69 | male | perihilar | poor | yes | III | 400 | yes |
| p45 | 279 | die | 57 | male | distal | poor | yes | IIB | 300 | yes |
| p46 | 248 | die | 66 | male | distal | well | no | IIB | 300 | yes |
| p47 | 1860 | live | 66 | female | perihilar | well | no | IA | 100 | yes |
| p48 | 1116 | die | 59 | female | perihilar | poor | yes | III | 200 | yes |
| p49 | 620 | die | 65 | male | perihilar | poor | no | IIA | 100 | yes |
| p50 | 155 | die | 66 | male | distal | poor | no | III | 200 | no |
| p51 | 1519 | die | 68 | female | perihilar | poor | yes | III | 200 | yes |
| p52 | 1860 | live | 42 | female | distal | poor | no | IIA | 1000 | yes |
| p53 | 527 | die | 48 | male | distal | poor | no | IIA | 50 | yes |
| p54 | 1023 | die | 58 | male | distal | poor | yes | IIB | 400 | yes |
| p55 | 434 | die | 61 | male | perihilar | poor | yes | IIB | 500 | yes |
| p56 | 341 | die | 58 | male | distal | poor | yes | IIB | 1000 | yes |
| p57 | 124 | die | 47 | male | distal | poor | yes | IIB | 200 | no |
| p58 | 806 | die | 64 | female | perihilar | well | no | IB | 500 | yes |
| p59 | 961 | die | 74 | male | perihilar | well | no | IA | 500 | no |
| p60 | 589 | die | 56 | female | perihilar | poor | yes | IIB | 700 | yes |
| p61 | 1860 | live | 75 | male | perihilar | poor | no | IB | 200 | yes |
| p62 | 465 | die | 63 | male | perihilar | poor | yes | III | 700 | yes |
| p63 | 1860 | live | 67 | male | distal | well | no | IB | 100 | yes |
| p64 | 837 | die | 64 | male | perihilar | well | no | IIA | 50 | yes |
| p65 | 1209 | die | 53 | male | distal | well | no | IIA | 150 | yes |
| p66 | 1860 | live | 55 | male | perihilar | well | no | IB | 100 | yes |
| p67 | 465 | die | 66 | male | perihilar | well | no | IIA | 600 | yes |
| p68 | 620 | die | 46 | male | perihilar | well | no | IB | 1500 | yes |
| p69 | 217 | die | 60 | female | distal | well | no | IB | 300 | yes |
| p70 | 1860 | die | 60 | male | perihilar | well | no | IIA | 200 | yes |
| p71 | 713 | die | 54 | male | distal | well | yes | IIB | 100 | yes |
| p72 | 1860 | live | 62 | male | perihilar | well | no | IB | 150 | yes |
| p73 | 1023 | die | 64 | female | distal | well | no | IIA | 800 | yes |
| p74 | 403 | die | 75 | male | perihilar | well | no | IB | 100 | no |
| p75 | 682 | die | 46 | female | perihilar | poor | no | IB | 150 | yes |
| p76 | 1860 | live | 54 | male | distal | well | no | IB | 450 | yes |
| p77 | 589 | die | 59 | female | perihilar | poor | no | IB | 100 | yes |
| p78 | 1860 | live | 53 | male | distal | well | no | IB | 200 | yes |
| p79 | 1860 | live | 65 | male | perihilar | well | no | IB | 100 | yes |
| p80 | 1426 | die | 57 | male | distal | well | no | IIA | 300 | yes |
| p81 | 1860 | live | 65 | female | perihilar | poor | no | IIA | 1100 | yes |
| p82 | 1488 | live | 53 | female | perihilar | well | no | IB | 150 | yes |
| p83 | 434 | die | 64 | male | perihilar | well | no | IIA | 300 | yes |
| p84 | 775 | die | 71 | female | distal | well | no | IIA | 200 | yes |
| p85 | 651 | die | 55 | male | distal | well | yes | IIB | 500 | yes |
| p86 | 372 | die | 64 | male | perihilar | poor | no | IIA | 300 | yes |
| p87 | 496 | die | 64 | female | perihilar | poor | no | IA | 50 | yes |
| p88 | 1612 | live | 64 | male | perihilar | well | no | IIA | 200 | yes |
| p89 | 1612 | live | 70 | female | distal | poor | no | IIA | 500 | yes |
| p90 | 372 | die | 56 | female | distal | poor | no | IIA | 200 | yes |
| p91 | 1550 | live | 48 | male | perihilar | poor | no | IIA | 300 | yes |
| p92 | 589 | die | 67 | male | distal | well | no | IIA | 400 | yes |
| p93 | 1519 | live | 56 | male | distal | poor | no | IIA | 600 | yes |
| p94 | 1519 | live | 67 | male | distal | well | no | III | 300 | yes |
| p95 | 651 | die | 58 | male | perihilar | poor | no | IA | 200 | yes |
| p96 | 713 | die | 48 | male | distal | well | no | IIA | 400 | yes |
| p97 | 310 | die | 44 | male | distal | well | yes | II | 200 | yes |
| p98 | 1488 | live | 68 | male | distal | well | no | IIA | 300 | yes |
| p99 | 1426 | live | 65 | female | perihilar | well | no | IIA | 200 | yes |
| p100 | 1426 | live | 57 | male | distal | poor | yes | II | 500 | yes |
| p101 | 124 | die | 70 | female | distal | poor | no | IIA | 200 | yes |
| p102 | 372 | die | 57 | male | perihilar | well | no | IIA | 200 | yes |
| p103 | 341 | die | 68 | male | perihilar | well | no | IIA | 600 | yes |
| p104 | 310 | die | 64 | female | perihilar | poor | no | IIA | 2700 | yes |
| p105 | 279 | die | 61 | male | perihilar | poor | no | IA | 700 | yes |
| p106 | 1271 | live | 60 | male | distal | poor | no | IIA | 300 | yes |
| p107 | 1271 | live | 46 | male | perihilar | well | no | IA | 300 | yes |
| p108 | 186 | die | 54 | male | perihilar | poor | yes | III | 30 | no |
| p109 | 310 | die | 52 | male | perihilar | poor | no | IA | 400 | yes |
| p110 | 1333 | live | 64 | male | distal | well | no | IIA | 400 | yes |
| p111 | 682 | die | 53 | male | perihilar | well | no | IIA | 200 | no |
| p112 | 744 | die | 59 | male | perihilar | well | no | IIA | 1500 | yes |
| p113 | 496 | die | 69 | female | perihilar | well | no | IIA | 100 | yes |
| p114 | 1085 | die | 69 | male | perihilar | poor | no | IIA | 200 | yes |
| p115 | 248 | die | 36 | male | perihilar | poor | no | IA | 30 | yes |
| p116 | 310 | die | 64 | male | perihilar | well | no | IA | 400 | yes |
| p117 | 434 | die | 68 | male | perihilar | poor | no | IIA | 60 | yes |
| p118 | 1116 | die | 64 | male | perihilar | well | no | IA | 200 | yes |
| p119 | 1240 | live | 58 | male | distal | well | no | IIA | 100 | yes |
| p120 | 1240 | live | 75 | female | perihilar | well | no | IIA | 300 | yes |
| p121 | 1240 | live | 53 | male | distal | poor | no | IIA | 600 | yes |
| p122 | 1240 | live | 61 | female | perihilar | poor | yes | III | 300 | yes |
| p123 | 1240 | live | 68 | male | distal | well | no | IIA | 800 | yes |
| p124 | 372 | die | 63 | male | perihilar | well | no | IIA | 100 | yes |
| p125 | 899 | die | 53 | female | perihilar | well | no | IA | 300 | yes |
| p126 | 899 | die | 68 | female | perihilar | well | no | IA | 200 | yes |
| p127 | 1209 | live | 72 | male | distal | well | no | IIA | 200 | yes |
| p128 | 186 | die | 59 | male | distal | well | no | III | 500 | yes |
| p129 | 589 | die | 49 | female | distal | well | no | IIA | 500 | yes |
| p130 | 651 | die | 65 | male | perihilar | well | no | IIA | 500 | yes |
| p131 | 961 | die | 59 | male | perihilar | well | no | IIA | 200 | yes |
| p132 | 1178 | live | 65 | female | perihilar | poor | yes | III | 100 | yes |
| p133 | 1147 | live | 66 | male | perihilar | well | no | III | 200 | yes |
| p134 | 248 | die | 65 | male | distal | poor | no | III | 500 | yes |
| p135 | 837 | die | 46 | male | distal | well | no | IIA | 500 | yes |
| p136 | 341 | die | 61 | male | perihilar | poor | no | III | 400 | yes |
| p137 | 279 | die | 61 | male | perihilar | poor | yes | III | 400 | yes |
| p138 | 1085 | live | 59 | male | distal | well | no | IIA | 100 | yes |
| p139 | 1023 | live | 73 | male | perihilar | well | no | IIA | 150 | yes |
| p140 | 1023 | live | 66 | male | distal | well | no | IIA | 50 | yes |
