# Supplementary figures and images for: Preoperative peripheral blood inflammatory markers especially the fibrinogen-to-lymphocyte ratio and novel FLR-N score predict the prognosis of patients with early-stage resectable extrahepatic cholangiocarcinoma
Source: Front Oncol. 2022 Oct 31;12:1003845. doi: 10.3389/fonc.2022.1003845 (PMC9659886; doi:10.3389/fonc.2022.1003845)

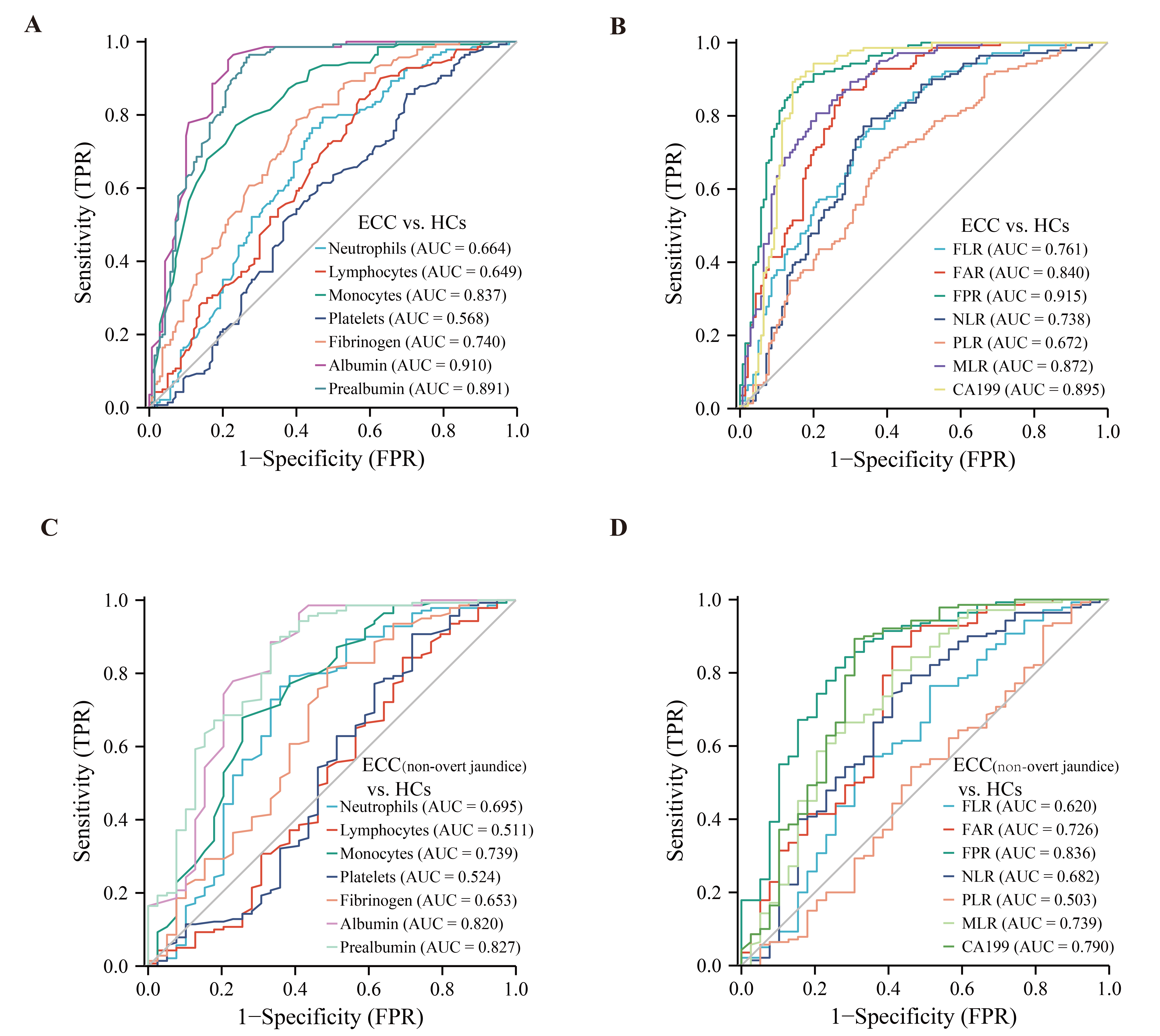

Supplement: Supplementary file 2 [file Image_1.tif]

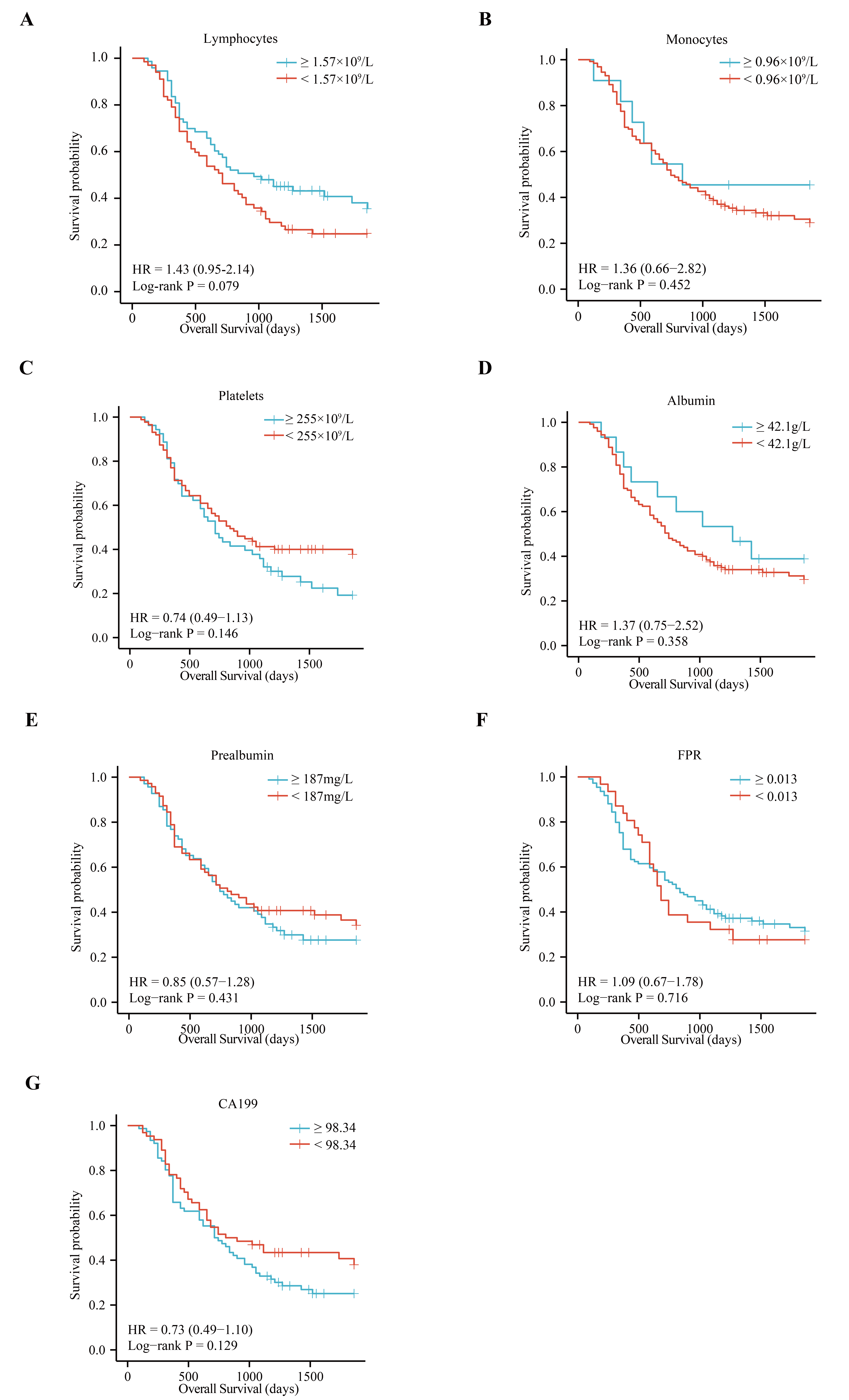

Supplement: Supplementary file 3 [file Image_2.tif]

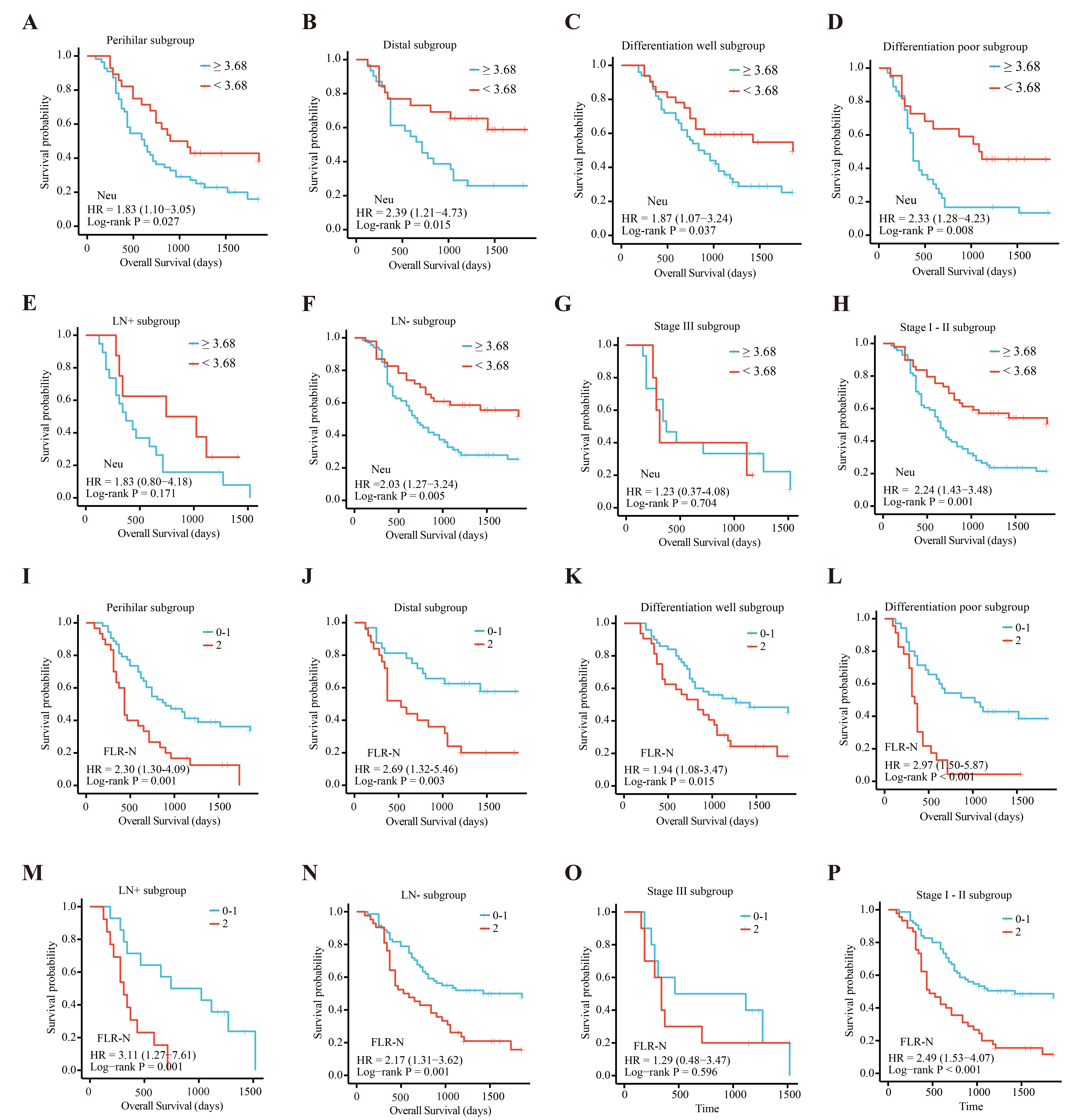

Supplement: Supplementary file 4 [file Image_3.tif]

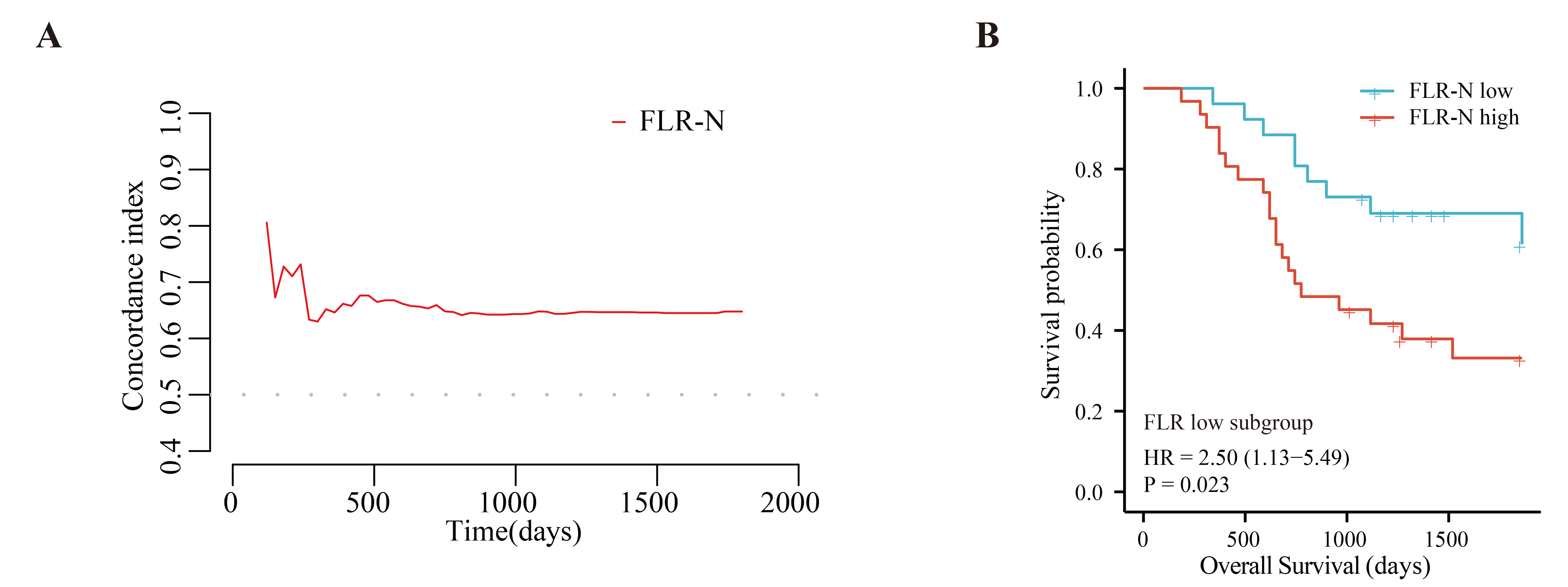

Supplement: Supplementary file 5 [file Image_4.tif]
